# miR-149-3p targeting *TMPRSS4* regulates the sensitivity to cisplatin to inhibit the progression of lung cancer

**DOI:** 10.17305/bb.2024.11163

**Published:** 2024-10-01

**Authors:** Beibei Qin, Dongfang Tang, Mingzhi Zhang

**Affiliations:** 1 Department of Oncology, The First Affiliated Hospital of Zhengzhou University, Zhengzhou, Henan Province, China; 2 Department of Oncology, LuoYang Central Hospital Affiliated to Zhengzhou University, Luoyang, Henan Province, China; 3 Department of Thoracic Surgery, Huadong Hospital Affiliated to Fudan University, Jing’an District, Shanghai, China

**Keywords:** miR-149-3p, lung cancer, *TMPRSS4*, DDP sensitivity

## Abstract

Lung cancer cells tend to develop resistance to cisplatin (DDP) during continuous chemotherapy, making it crucial to improve DDP sensitivity to enhance therapeutic outcomes. The levels of miR-149-3p in lung tissues and cells, as well as the biological behaviors of lung cancer cells, were analyzed. H446/DDP and A549/DDP cell lines were established to investigate how miR-149-3p affects lung cancer cells’ sensitivity to DDP. Bioinformatics analysis predicted transmembrane serine protease 4 (*TMPRSS4*) as a downstream target of miR-149-3p, which was subsequently confirmed. Western blot analysis was used to examine proteins related to migration, invasion, apoptosis, and TMPRSS4 expression. Additionally, a subcutaneous graft tumor model in nude mice was created to assess the impact of miR-149-3p on tumor growth. In lung cancer tissues and cells, miR-149-3p expression was reduced, while TMPRSS4 expression was elevated. Overexpression of miR-149-3p inhibited cancer progression, promoted apoptosis, and enhanced the chemosensitivity of lung cancer cells to DDP. Moreover, miR-149-3p negatively regulated *TMPRSS4*, reducing malignancy-associated characteristics of lung cancer cells and further improving their DDP sensitivity. In vivo, high miR-149-3p expression increased the chemosensitivity of cancer cells. In conclusion, miR-149-3p suppresses the aggressive progression of lung cancer by directly downregulating *TMPRSS4* and enhances the responsiveness of lung cancer cells to DDP.

## Introduction

Lung cancer, a frequently diagnosed condition, affected 2.2 million people and resulted in 1.8 million deaths worldwide in 2020 [1, 2]. Lung cancer is divided into two major types: small cell lung cancer (SCLC) and non-small cell lung cancer (NSCLC) [3, 4]. SCLC accounts for approximately 15% of lung cancer cases, while NSCLC makes up 85%. NSCLC has several subtypes [5, 6] and is highly malignant, progressing rapidly. Consequently, most patients are diagnosed at a late stage, for which chemotherapy, particularly platinum-based treatments, is often the preferred option [7, 8]. However, prolonged use of cis-diamminedichloroplatinum (DDP) in chemotherapy leads to drug resistance in lung cancer cells, reducing its therapeutic efficacy [9, 10]. Thus, enhancing the sensitivity of lung cancer cells to DDP is crucial.

MicroRNAs (miRNAs), approximately 22–24 nucleotides long, belong to the category of non-coding RNAs. Many studies have shown that miRNAs play key roles in regulating various cellular processes, including apoptosis, proliferation, and disease progression [11, 12]. miRNAs target specific genes by base pairing and binding to the target gene’s 3′-UTR, resulting in mRNA degradation or translation inhibition [13]. Depending on their regulation in tumors, miRNAs can function as oncogenes or tumor suppressors [14]. miR-149-3p, located on chromosome 2q37.3, is encoded by the precursor gene *miR-149*. Numerous studies have demonstrated that dysregulation of miR-149-3p is associated with pathological processes in cancer, including tumor cell proliferation, metastasis, and apoptosis [15, 16]. Shen et al. [17] found that miR-149-3p overexpression reduced the proliferation and viability of oral squamous cell carcinoma cells. Another study [18] demonstrated a link between miR-149-3p and DDP resistance in ovarian cancer cells. However, its role in lung cancer progression and its impact on DDP sensitivity remain unclear.

Transmembrane serine protease 4 (*TMPRSS4*), a type II transmembrane serine protease, is located on chromosome 11q23.3 (Gene ID: 56649). It is expressed in several human organs, including the esophagus, stomach, small intestine, colon, bladder, and kidney, though its physiological function is not well understood [19, 20]. Research indicates that TMPRSS4 is overexpressed in cancers such as prostate cancer, pancreatic ductal adenocarcinoma, and gastric cancer, and is associated with poor prognosis [21–23]. Importantly, overexpression of TMPRSS4 in lung cancer cells is linked to increased resistance to DDP and other standard chemotherapeutic agents [24]. However, the specific mechanisms by which TMPRSS4 mediates DDP resistance in lung cancer cells remain insufficiently studied.

This study first investigated miR-149-3p levels in lung cancer tissues and cells, examining the effects of miR-149-3p overexpression or silencing on the malignant behavior of lung cancer cells. DDP-resistant cells were then generated to assess their miR-149-3p levels. *TMPRSS4*, identified through multiple databases, was found to be a target of miR-149-3p. Finally, miR-149-3p and/or TMPRSS4 were overexpressed to observe changes in lung cancer cells, particularly DDP-resistant cells, to clarify the roles of miR-149-3p and TMPRSS4 in modulating DDP resistance in lung cancer, providing further insight for clinical treatment.

## Materials and methods

### Samples of clinical tissues

Tissue samples were obtained from 45 lung cancer patients admitted to LuoYang Central Hospital Affiliated with Zhengzhou University between January and November 2020. Cancerous tissue samples and adjacent normal lung tissue samples were collected during surgery, washed, and stored in liquid nitrogen for future use. All patients were treatment-naive, having not received radiotherapy or chemotherapy before surgery. Informed consent was obtained from all participants, and the study was approved by the Ethics Committee of LuoYang Central Hospital Affiliated with Zhengzhou University (Approval number: 2021K010).

Inclusion criteria: (1) age ≥ 18 years, any gender; (2) pathological confirmation of lung cancer; (3) provision of written informed consent; and (4) complete personal data. Exclusion criteria: (1) prior treatments, such as targeted therapy, chemotherapy, radiotherapy, or immunotherapy; (2) a history of other malignant tumors; (3) diagnosis of benign tumors; and (4) extrapulmonary metastasis.

### Cell treatments

A range of lung cancer cell lines, including A549, H1650, and H446, along with the normal lung epithelial BEAS-2B cell line, were obtained from the Shanghai Cell Bank, part of the Chinese Academy of Sciences. Cells were cultured using DMEM (11965118, Gibco, USA) supplemented with 1% double antibiotics (15140122, Gibco) and 10% fetal bovine serum (A5670701, Gibco) to maintain cell health and proliferation. Transfection experiments involved the use of miR-149-3p mimic (mimic, 2237813), miR-149-3p inhibitor (inhibitor, 2237847), miR-149-3p lentiviral vector (LV-miR-149-3p, 2237912), TMPRSS4 overexpression plasmid (pcDNA-TMPRSS4, 2237854), and their respective negative controls (mimic-NC, inhibitor-NC, and LV-NC), all purchased from Sangon Biotech (Shanghai, China). Transfections were conducted in H446 and A549 cells using the Lipofectamine 3000 (L3000150, Invitrogen) protocol.

### Formation of DDP-resistant cells

Following the methods of Fu et al. [25] and Monroe et al. [26], H446 and A549 cells were treated with increasing doses of DDP (0, 2, 4, and 6 µg/mL; D8810, Solarbio, Beijing, China). The treatment continued until the cells reached a stable growth and passage state under 6 µg/mL DDP. These resistant cells were then cultured in DMEM high-glucose medium (10%) at 37 ^∘^C with 5% CO_2_.

### qRT-PCR assay

Total RNA was extracted using Trizol Reagent (15596026, Invitrogen). Reverse transcription of 2 µg total RNA was carried out with AMV reverse transcriptase (2621, TAKARA, Tokyo, Japan). cDNA was amplified using SYBR Green qPCR Mix (CN830S, TAKARA, Tokyo, Japan), with U6 and GAPDH serving as internal controls.

- U6 primers: F: 5′-CTCGCTTCGGCAGCACA-3′, R: 5′-AACGCTTCACGAATTTGCGT-3′
- GAPDH primers: F: 5′-CATGTTCGTCATGGGTGTGAACC-3′, R: 5′-GGTCATGAGTCCTTCCACGATACC-3′
- miR-149-3p primers: F: 5′-TCGGCAGGAGGGAGGGACGGGGG-3′, R: 5′-GTAGCCCATGGGTTTTAGCCC-3′
- TMPRSS4 primers: F: 5′-GGATCACAGAGCCAGCATGT-3′, R: 5′-TTCGGGGAAGCTCTTGACAC-3′

### Cell Counting Kit-8 (CCK-8) assay

H446 and A549 cells (1.5×10^4^) were seeded in 96-well plates. After cell adherence, the medium was replaced. At 24, 48, and 72 h post-seeding, 10% CCK-8 diluted in 100 µL of complete medium (C0038, Beyotime, China) was added and cells were incubated at 37 ^∘^C for 2 h. Finally, the optical density (OD) at 450 nm was measured using a microplate reader.

### Carboxyfluorescein diacetate succinimidyl ester (CFSE) staining

A549 and H446 cells were prepared in PBS at a density of 1.0×10^6^ cells/mL. CFSE probe (C1031, Beyotime) was added to a 1 mL cell suspension to a final concentration of 5 µM. The mixture was incubated at 37 ^∘^C for 10 min. Staining was stopped with 5× complete medium, and cells were washed, resuspended in PBS, and placed into a 96-well plate for observation under a fluorescence microscope. After 48 h of treatment with plasmids or 8 µg/mL of DDP, cell proliferation was assessed by flow cytometry (FCM) (BD FACSCaliburTM, USA).

### Transwell assay

Matrigel (354230, Corning, Tewksbury, MA, USA) was diluted in serum-free high-glucose DMEM and added to the upper Transwell chamber (100 µL), which was then incubated overnight. The next day, the remaining liquid was removed, and the membrane was hydrated with serum-free DMEM. Cells (1.0×10^5^ cells/mL) were added to the upper chamber (200 µL), and 800 µL of complete medium with or without DDP was placed in the lower chamber. After 48 h of incubation, the cells were fixed, stained with crystal violet (C0775, Sigma-Aldrich), and washed twice with PBS. Random fields of view were photographed under a fluorescence microscope (DM IL LED, Leica, Heidelberg, Germany), and the number of invaded cells was counted.

### Wound healing assay

Cells were digested with trypsin (25200114, Gibco) and adjusted to a concentration of 3.0×10^5^ cells/mL. Cells (1 mL) were seeded into a 6-well plate with marked horizontal lines. After the cells reached 80% confluence, the medium was removed, and a wound was created by scratching the cell monolayer with a 20 µL sterile pipette tip in a crisscross pattern. Cells were rinsed with PBS, and serum-free medium, with or without DDP, was added. Scratch closure was monitored at 0 and 48 h, and the cell migration rate was calculated using Image J software (version 1.54h, Wavne Resband, National Institute of Mental Health, USA).

### FCM assay

H446 and A549 cells in the log phase were washed twice with PBS. Then, 1% Annexin-V-FITC (HY-K1073, MedChemExpress, Monmouth Junction, NJ, USA) and propidium iodide (ST1569, Beyotime), diluted in binding buffer, were added. The samples were incubated in the dark for 15 min and transferred to FCM tubes for analysis within 1 h to assess apoptotic status.

### Bioinformatics analysis

Potential downstream targets of miR-149-3p were identified using bioinformatics tools such as GEPIA (http://gepia.cancer-pku.cn/), TargetScanHuman 8.0 (https://www.targetscan.org/vert_80/), miRDB (https://mirdb.org/index.html), and miRWalk (http://mirwalk.umm.uni-heidelberg.de/). These tools helped identify *TMPRSS4* as a candidate target, which was subsequently validated experimentally.

### Dual-luciferase reporter assay

Wild-type (WT) and mutant (MUT) *TMPRSS4* 3′UTR sequences were obtained from Sangon Biotech (Shanghai, China). H446 and A549 cells were transfected using Lipofectamine 3000 with *TMPRSS4* 3′ UTR-WT & mimic-NC, *TMPRSS4* 3′ UTR-WT & mimic, *TMPRSS4* 3′ UTR-MUT & mimic-NC, or *TMPRSS4* 3′ UTR-MUT & mimic. After 48 h, luciferase activity was measured using a dual-luciferase assay kit (D0010, Solarbio).

### Tumor formation in nude mice

Athymic mice were divided into four groups (five mice per group). Two groups were injected subcutaneously with A549 cells expressing LV-miR-149-3p (1.5×10^6^ cells/mouse), and the other two groups with LV-NC-expressing cells (1.5×10^6^ cells/mouse). On the 7th day, one LV-miR-149-3p group and one LV-NC group received an intraperitoneal injection of 8 µg/mL DDP, repeated every two weeks [27]. Tumor size was measured on days 7, 14, 21, 28, and 35. On day 35, the mice were sacrificed, and tumors were excised for imaging.

### Immunohistochemistry

Tumor sections (4–5-µm thick) were prepared and underwent xylene dewaxing (247642, Sigma), antigen retrieval, and incubation with 3% H_2_O_2_ for 25 min. After blocking with 5% bovine serum albumin (V900933, Sigma), sections were incubated with primary antibody against Ki67 (ab16667, 1:200, Abcam, Cambridge, MA, USA) at 37 ^∘^C for 90 min. IgG secondary antibody (31460, 1:10000, Invitrogen) was applied, followed by DAB (DA1010, Solarbio) development. The sections were counterstained with Mayer hematoxylin (MHS 16, Sigma-Aldrich) and visualized under a fluorescence microscope.

### TUNEL staining

Dewaxed tumor sections were hydrated in ethanol (100%, 95%, 75%, 50%) for 5 min each, followed by incubation with 20 µg/mL protease K (ST532, Beyotime) for 30 min. TUNEL detection solution (C1086, Beyotime) was applied for 1.5 h, followed by DAPI (D9542, Sigma-Aldrich) staining for 10 min. Fluorescence was captured with an inverted microscope.

### Western blot assay

Proteins were extracted using RIPA buffer (P0013B, Beyotime) and quantified using a BCA kit (P0012, Beyotime). Proteins were separated by gel electrophoresis and transferred to a membrane. After blocking, membranes were incubated overnight at 4 ^∘^C with primary antibodies: TMPRSS4 (PA5-18871, 1:1000, Invitrogen), MMP-2 (ab97779, 1:500, Abcam), MMP-9 (ab76003, 1:300, Abcam), Ki67 (1:1000), Cleaved-caspase 9 (PA5-105271, 1:2000, Invitrogen), Cleaved-caspase 3 (PA5-30622, 1:2000, Invitrogen), Bax (ab53154, 1:100, Abcam), and Bcl-2 (ab59348, 1:500, Abcam). After washing, membranes were incubated with secondary antibody IgG (31460, 1:10000, Invitrogen) for 1.5 h. Chemiluminescence reagent ECL (34580, Thermo Fisher Scientific) was applied, and protein bands were scanned using a gel imaging system (iBright CL1500, Invitrogen). GAPDH (MA1-16757, 1:1000, Invitrogen) served as the loading control, and band intensities were analyzed with Image J software.

### Ethical statement

This study was approved by the Ethics Committee of LuoYang Central Hospital Affiliated with Zhengzhou University (Approval No. 2021K010). Animal experiments were conducted following approval from the Experimental Animal Ethics Committee of LuoYang Central Hospital Affiliated with Zhengzhou University (Approval No. 2022K142).

### Data processing and analysis

All experiments were repeated at least three times, and results are presented as mean ± standard deviation. Data were analyzed using GraphPad Prism 9.0, and graphs were generated using the same software. Statistical significance was determined by Student’s *t*-test for two-sample comparisons and ANOVA for multiple group comparisons. A *P* value < 0.05 was considered significant, with non-significant differences marked as “ns”.

## Results

### miR-149-3p levels in lung cancer cells and tissues

**Figure 1.**
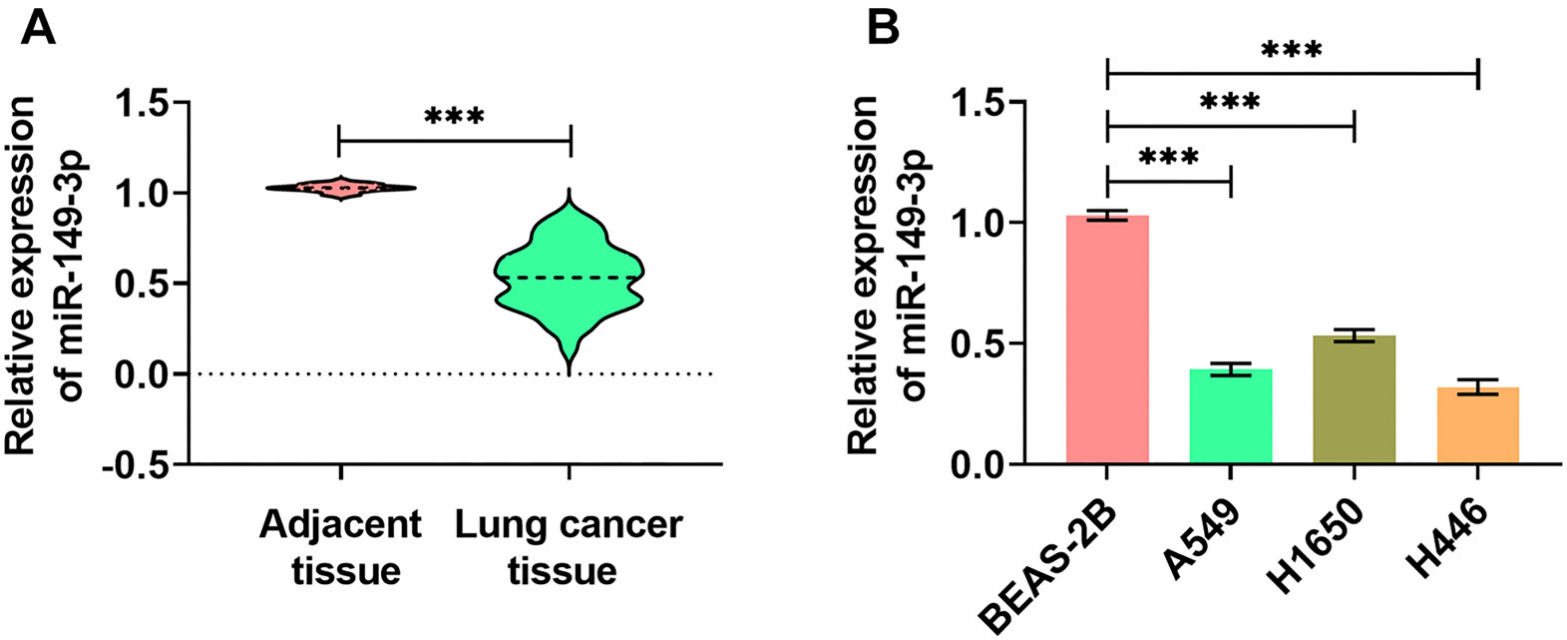
**miR-149-3p contents in tissue and cell**. (A) qRT-PCR results showing miR-149-3p levels in lung cancer tissues versus adjacent normal tissues (*n* ═ 45); (B) qRT-PCR results showing miR-149-3p levels in lung cancer cell lines (A549, H1650, H446) compared to normal cells (BEAS-2B). (***P < 0.001).

qRT-PCR analysis revealed that miR-149-3p levels were significantly higher in para-cancerous normal tissues compared to lung cancer tissues (Figure 1A). Additionally, miR-149-3p levels were significantly lower in lung cancer cell lines (H446, A549, and H1650), particularly in H446 and A549, when compared to normal lung epithelial cells (BEAS-2B) (Figure 1B). Based on these results, H446 and A549 cells were selected for further experiments.

**Figure 2.**
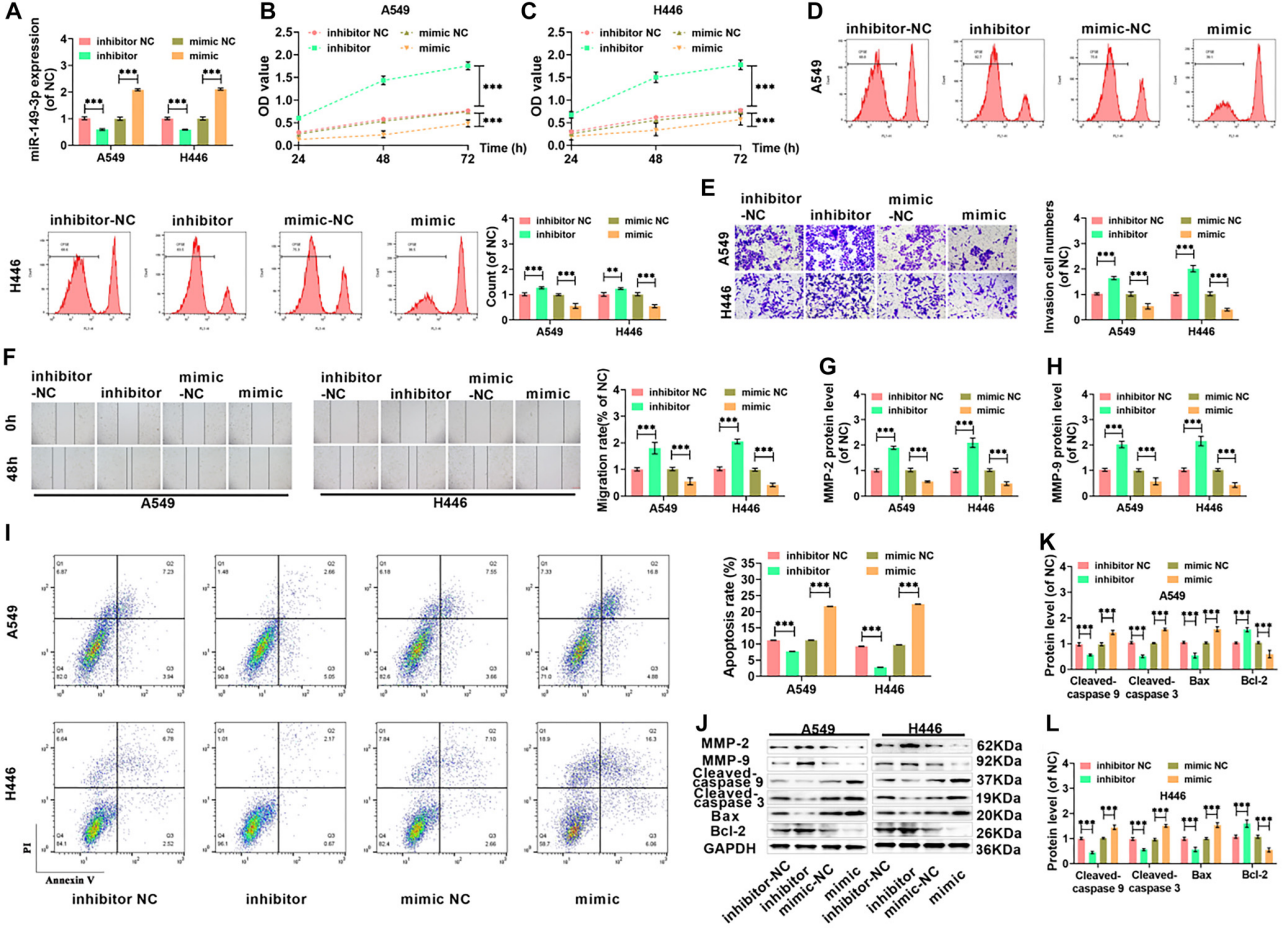
**miR-149-3p can inhibit proliferation, invasion, and migration and promoted apoptosis of lung cancer cells**. (A) qRT-PCR was used to determine miR-149-3p levels in A549 and H446 cells following silencing or overexpression of the miRNA; (B and C) The proliferative capacity of A549 and H446 cells after miR-149-3p silencing or overexpression was assessed; (D) Cell proliferation in A549 and H446 cells was further analyzed using CFSE staining, with data obtained via FCM; (E) The invasive capabilities of the cells post-treatment were measured using the Transwell assay (20×, bar ═ 100 µm), while their migratory abilities were assessed using the wound healing assay (10×, bar ═ 200 µm), with migration rates calculated accordingly (F); (G and H) Western blot analysis was used to quantify MMP-2 and MMP-9 protein levels in response to miR-149-3p silencing or overexpression in both cell lines; (I) The apoptotic rates of lung cancer cells with altered miR-149-3p expression were determined via flow cytometry; (J) Western blot analysis of cleaved caspase-9, cleaved caspase-3, Bax, Bcl-2, MMP-2, and MMP-9 proteins in A549 and H446 cells; (K and L) The levels of cleaved caspase-9, cleaved caspase-3, Bax, and Bcl-2 proteins were further measured following miR-149-3p modulation in both cell lines. (***P < 0.001). miRNA: MicroRNA; FCM: Flow cytometry; CFSE: Carboxyfluorescein succinimidyl ester.

**Figure 3.**
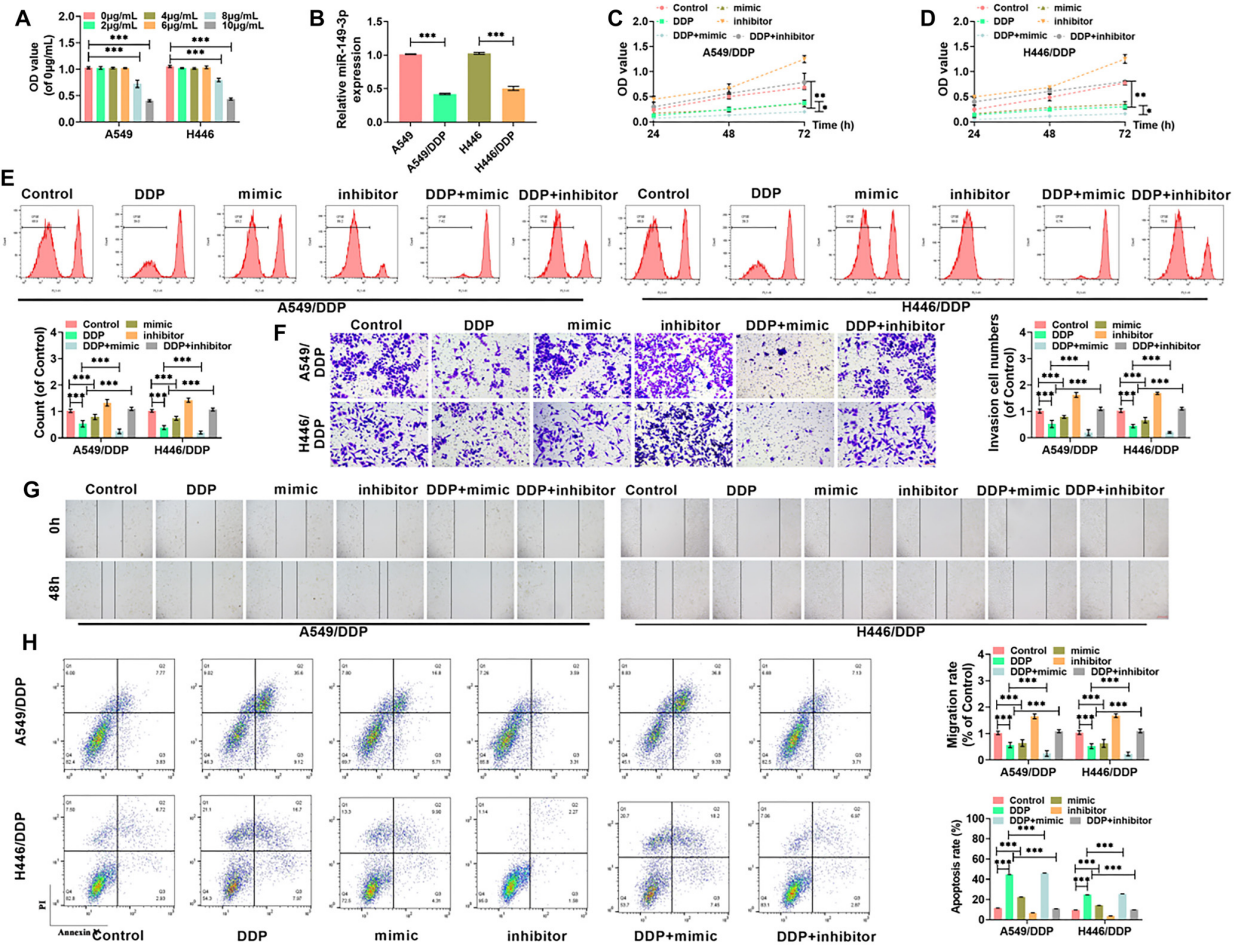
**miR-149-3p enhances the sensitivity of lung cancer cells to DDP, suppresses cell proliferation, invasion, and migration, and promotes cell apoptosis.** (A) The viability of lung cancer cells was measured following treatment with different concentrations of DDP; (B) Expression levels of miR-149-3p in A549, A549/DDP, H446, and H446/DDP cells; (C and D) The proliferative capacity of A549/DDP and H446/DDP cells; (E) Cell proliferation in A549/DDP and H446/DDP cells, as measured by FCM; (F) Transwell assay assessing the number of invading cells after different treatments (20×, bar ═ 100 µm); (G) Migration rates of A549/DDP and H446/DDP cells were calculated (10×, bar ═ 200 µm); (H) FCM was used to determine the extent of apoptosis. (*P < 0.05, **P < 0.01, ***P < 0.001). FCM: Flow cytometry; DDP: Diamminedichloroplatinum.

### miR-149-3p inhibits cell proliferation, invasion, and migration, and promotes apoptosis in lung cancer cells

H446 and A549 cells were transfected with either a miR-149-3p inhibitor or mimic, and qRT-PCR confirmed the modulation of miR-149-3p expression (Figure 2A). Silencing miR-149-3p significantly increased the viability of both A549 and H446 cells, while overexpression of miR-149-3p reduced cell viability (Figure 2B and 2C). CFSE fluorescence analysis showed that silencing miR-149-3p enhanced cell proliferation, while overexpression inhibited it (Figure 2D). The Transwell assay demonstrated that miR-149-3p silencing increased cell invasion, while overexpression reduced it (Figure 2E). Wound healing assays revealed that miR-149-3p silencing enhanced migration, while overexpression decreased it (Figure 2F). Western blot results showed that silencing miR-149-3p upregulated the expression of MMP-2 and MMP-9, while overexpression reduced these proteins (Figure 2G and 2H). FCM indicated that silencing miR-149-3p reduced apoptosis, whereas overexpression significantly promoted apoptosis (Figure 2I). Consistent with these findings, miR-149-3p silencing reduced the expression of pro-apoptotic proteins (Bax, Cleaved-caspase 9, and Cleaved-caspase 3) while increasing the anti-apoptotic protein Bcl-2; overexpression had the opposite effect (Figure 2J–2L).

**Figure 4.**
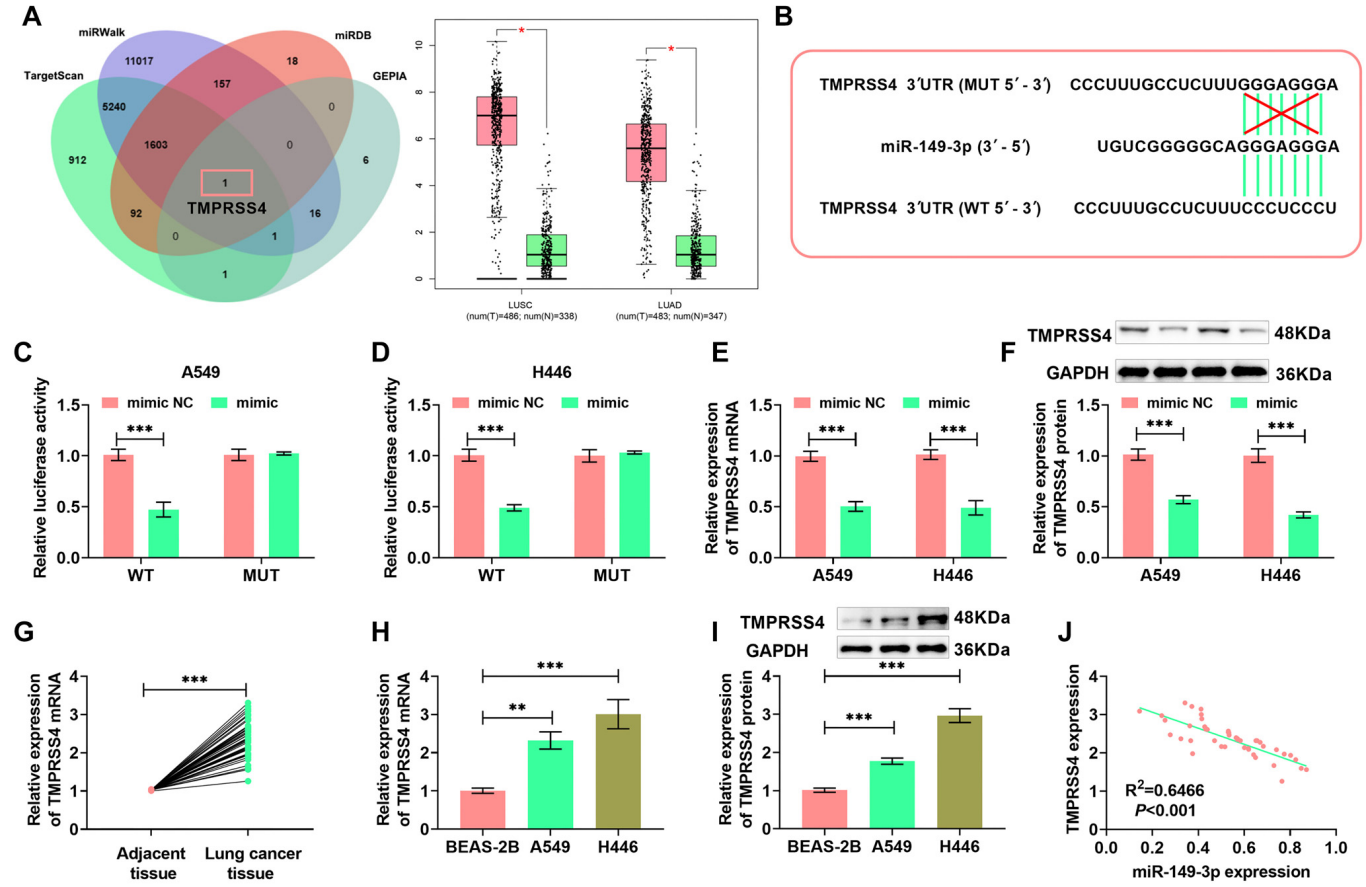
**Screening and verification of target gene for miR-149-3p**. (A) Screening of *TMPRSS4* and its expression in lung cancer; (B) Predicting the interaction sequences between miR-149-3p and *TMPRSS4* using TargetScan; (C and D) Results from the dual-luciferase assay revealed the regulatory relationship between miR-149-3p and *TMPRSS4*, emphasizing the miRNA’s targeting efficiency; (E) Results from qRT-PCR analysis of *TMPRSS4* mRNA expression; (F) Results from Western blot analysis of TMPRSS4 protein expression; (G and H) qRT-PCR results of *TMPRSS4* mRNA levels across various tissues and cells; (I) Western blot results showing TMPRSS4 protein levels in different cell types; (J) The linear correlation between miR-149-3p and *TMPRSS4* mRNA. (**P < 0.01, ***P < 0.001). miRNA: MicroRNA.

### miR-149-3p enhanced the sensitivity of lung cancer cells to cisplatin, inhibited cell proliferation, invasion, and migration, and promoted cell apoptosis

Treatment with 6 µg/mL DDP caused no significant change in the viability of H446 and A549 cells, whereas treatment with 8 µg/mL and 10 µg/mL DDP led to a sharp decline in cell viability after 48 h of incubation. This indicated that H446 and A549 cells had become resistant to 6 µg/mL DDP (Figure 3A). As shown by the qRT-PCR assay, miR-149-3p levels were markedly lower in H446/DDP and A549/DDP cells compared to normal H446 and A549 cells (Figure 3B). Following 48 h of exposure to 8 µg/mL DDP, A549/DDP and H446/DDP cells were transfected with either a miR-149-3p inhibitor or mimic, and cell viability was reassessed. DDP significantly reduced cell viability, with miR-149-3p overexpression enhancing DDP’s inhibitory effect, while miR-149-3p silencing weakened it (Figure 3C and 3D). After DDP treatment, the number of CFSE-positive cells significantly decreased, with miR-149-3p overexpression further suppressing proliferation, whereas miR-149-3p silencing partially reversed this effect (Figure 3E). DDP treatment also reduced cell invasion (Figure 3F) and mobility (Figure 3G), with miR-149-3p overexpression intensifying this inhibition while silencing miR-149-3p had the opposite effect. Furthermore, DDP significantly induced apoptosis, with miR-149-3p overexpression enhancing this effect, whereas silencing miR-149-3p reduced apoptosis (Figure 3H).

### Screening of miR-149-3p’s target gene

Using miRDB, GEPIA, miRWalk, and TargetScanHuman 8.0 databases to identify downstream target genes, *TMPRSS4* was identified as a target gene of miR-149-3p. As shown in the GEPIA database, TMPRSS4 expression was higher in lung cancer tissues compared to normal tissues (Figure 4A). The sequence pairing between miR-149-3p and *TMPRSS4* was predicted using TargetScan and is shown in Figure 4B. A dual-luciferase reporter assay confirmed that miR-149-3p significantly suppressed the luciferase signal in *TMPRSS4* WT but had no effect on *TMPRSS4* MUT (Figure 4C and 4D). Additionally, miR-149-3p overexpression reduced both TMPRSS4 protein and mRNA levels in H446 and A549 cells, further validating the targeted and negative regulation of TMPRSS4 expression by miR-149-3p (Figure 4E and 4F). TMPRSS4 protein and mRNA levels, as assessed by Western blot and qRT-PCR, were consistent with the GEPIA database findings, showing that TMPRSS4 is highly expressed in lung cancer tissues and cells (Figure 4G–4I). The linear regression analysis of TMPRSS4 and miR-149-3p expression (Figure 4J) indicated that miR-149-3p negatively regulates TMPRSS4 production.

**Figure 5.**
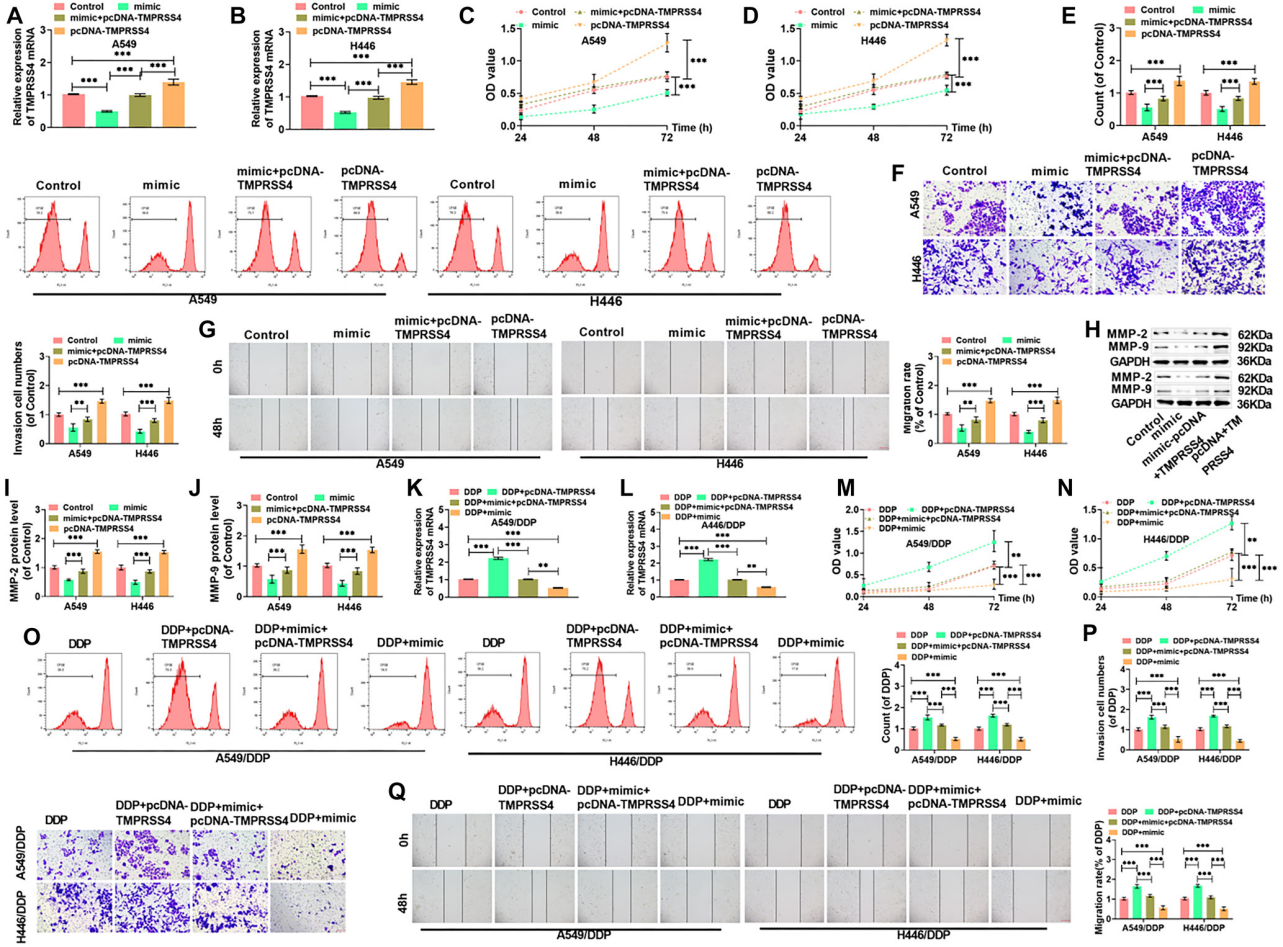
**TMPRSS4 can partially attenuate the effects of miR-149-3p on the sensitivity to cisplatin enhanced, and the cell proliferation, invasion, and migration inhibited of lung cancer cells**. (A and B) *TMPRSS4* mRNA levels in A549 and H446 cells; (C and D) Proliferative capacity of A549 and H446 cells; (E) Cell proliferation measured by FCM; (F) Invasion count of A549 and H446 cells after different treatments (20×, bar ═ 100 µm); (G) Migration ability and rate of A549 and H446 cells (10×, bar ═ 200 µm); (H–J) Western blot analysis of MMP-2 and MMP-9 protein levels in A549 and H446 cells; (K and L) *TMPRSS4* mRNA levels. (M and N) Proliferation capacity of A549/DDP and H446/DDP cells measured using the CCK-8 assay; (O) A549/DDP and H446/DDP cells were labeled with CFSE, and cell proliferation was assessed by FCM; (P) Invasion count of A549/DDP and H446/DDP cells after different treatments (20×, bar ═ 100 µm); (Q) Wound healing assay measured the migration of A549/DDP and H446/DDP cells (10×, bar ═ 200 µm). (**P < 0.01, ***P < 0.001). FCM: Flow cytometry; DDP: Diamminedichloroplatinum; CFSE: Carboxyfluorescein succinimidyl ester.

### *TMPRSS4* can partially attenuate the effects of miR-149-3p on cisplatin sensitivity, and on cell proliferation, invasion, and migration inhibition in lung cancer cells

H446 and A549 cells transfected with pcDNA-TMPRSS4 were tested through qRT-PCR. As a result, these cells exhibited significantly elevated TMPRSS4 levels, whereas those transfected with the mimic showed a much lower TMPRSS4 expression. This finding further confirms miR-149-3p's negative regulation of TMPRSS4 (Figures 5A–5B). Cell proliferation, assessed using the CCK-8 assay, indicated that the reduced proliferation capacity in H446 and A549 cells due to excessive miR-149-3p was somewhat restored by overexpressing TMPRSS4 (Figures 5C–5D). FCM assay results showed that overexpressing TMPRSS4 increased the number of CFSE-positive cells in both H446 and A549 cells, partially counteracting miR-149-3p's proliferation-inhibiting effects (Figure 5E). Furthermore, miR-149-3p overexpression significantly reduced invasion and migration in A549 and H446 cells, while TMPRSS4 overexpression partially enhanced both processes (Figures 5F–5G). After miR-149-3p overexpression, MMP-2 and MMP-9 levels were notably decreased in A549 and H446 cells, but TMPRSS4 overexpression partially reversed this effect (Figures 5H–5J).

Additionally, pcDNA-TMPRSS4 was transfected into H446/DDP and A549/DDP cells to explore the impact of TMPRSS4 overexpression on these cells. qRT-PCR results revealed that TMPRSS4 levels in H446/DDP and A549/DDP cells greatly decreased following miR-149-3p upregulation but increased with TMPRSS4 overexpression. However, this effect was somewhat counteracted by miR-149-3p overexpression (Figures 5K–5L). Overexpression of miR-149-3p reduced the proliferation capacity of A549/DDP and H446/DDP cells (Figures 5M and 5N), significantly decreased the number of CFSE-positive cells (Figure 5O), reduced the number of invading cells (Figure 5P), and lowered migration rates (Figure 5Q). However, these effects were mitigated by TMPRSS4 overexpression. Collectively, these results demonstrate that excessive TMPRSS4 enhances the resistance of lung cancer cells to DDP.

### *TMPRSS4* can partially attenuate the effects of miR-149-3p on apoptosis promotion and enhanced DDP sensitivity in lung cancer cells

**Figure 6.**
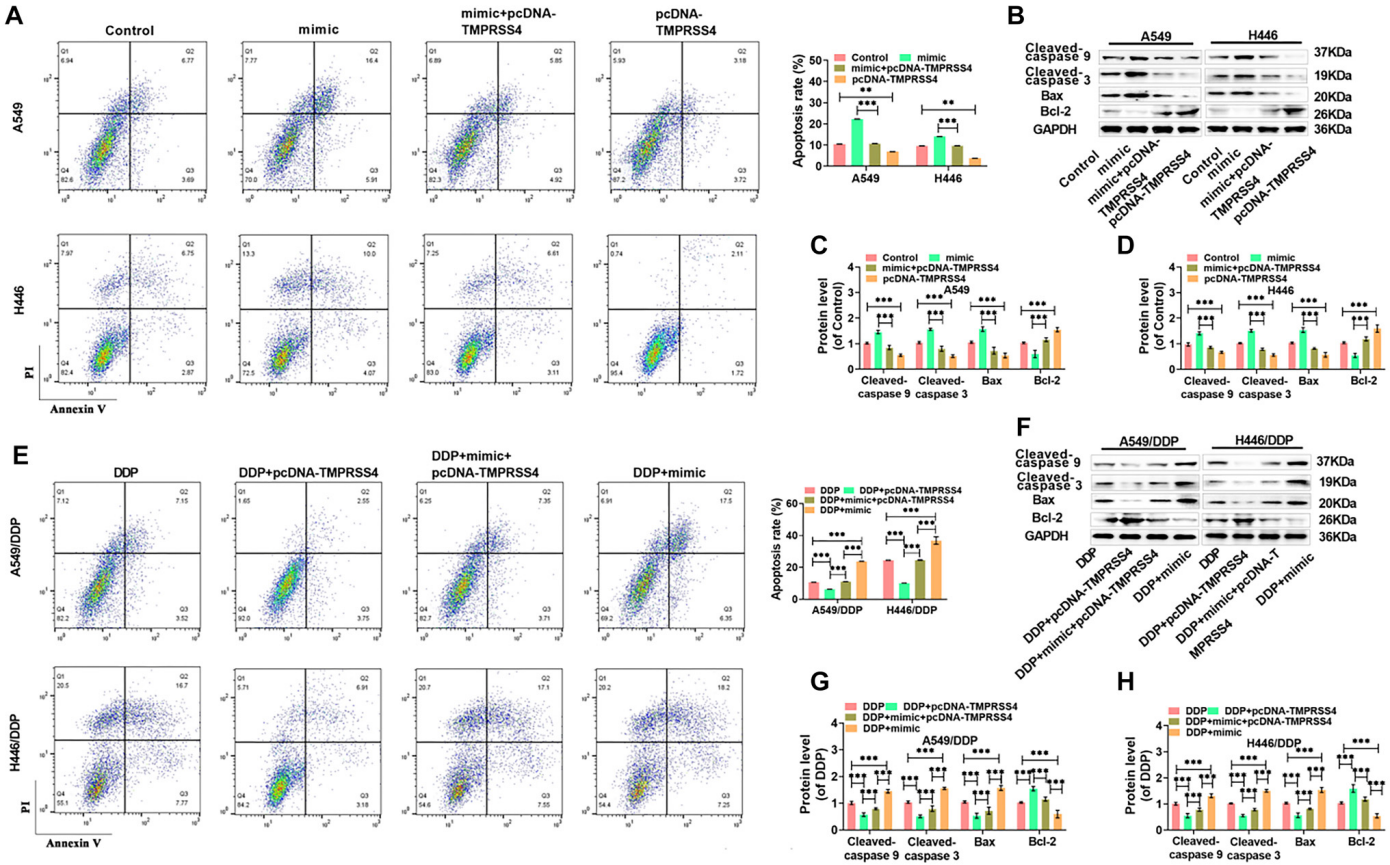
**TMPRSS4 can partially attenuate the effects of miR-149-3p on the sensitivity enhanced and apoptosis promoted of lung cancer cells**. (A) The apoptotic rate indicating the death rate of A549 and H446 cells; (B–D) Western blotting was performed to assess the levels of apoptosis-related proteins in A549 and H446 cells, including Cleaved-caspase 9, Cleaved-caspase 3, Bax, and Bcl-2; (E) Apoptosis rate of A549/DDP and H446/DDP cells; (F–H) Western blot analysis of Cleaved-caspase 9, Cleaved-caspase 3, Bax, and Bcl-2 expression in A549/DDP and H446/DDP cells. (**P < 0.01, ***P < 0.001). DDP: Diamminedichloroplatinum.

To determine whether miR-149-3p promotes apoptosis by targeting *TMPRSS4*, FCM revealed that miR-149-3p overexpression increased apoptosis in A549 and H446 cells, whereas TMPRSS4 overexpression reversed this effect (Figure 6A). miR-149-3p overexpression significantly increased the levels of Bax, Cleaved-caspase 9, and Cleaved-caspase 3 while reducing Bcl-2, an effect that was mitigated by TMPRSS4 overexpression, consistent with FCM results (Figure 6B–6D). In A549/DDP and H446/DDP cells, miR-149-3p overexpression also increased apoptosis and related proteins such as Bax, Cleaved-caspase 9, and Cleaved-caspase 3, while TMPRSS4 overexpression partially inhibited these effects (Figure 6E–6H). These results suggest that miR-149-3p enhances DDP sensitivity in lung cancer cells and promotes apoptosis, while TMPRSS4 reduces the effects of miR-149-3p and DDP, confirming that miR-149-3p enhances chemotherapeutic sensitivity by negatively regulating *TMPRSS4*.

### In vivo experiment

**Figure 7.**
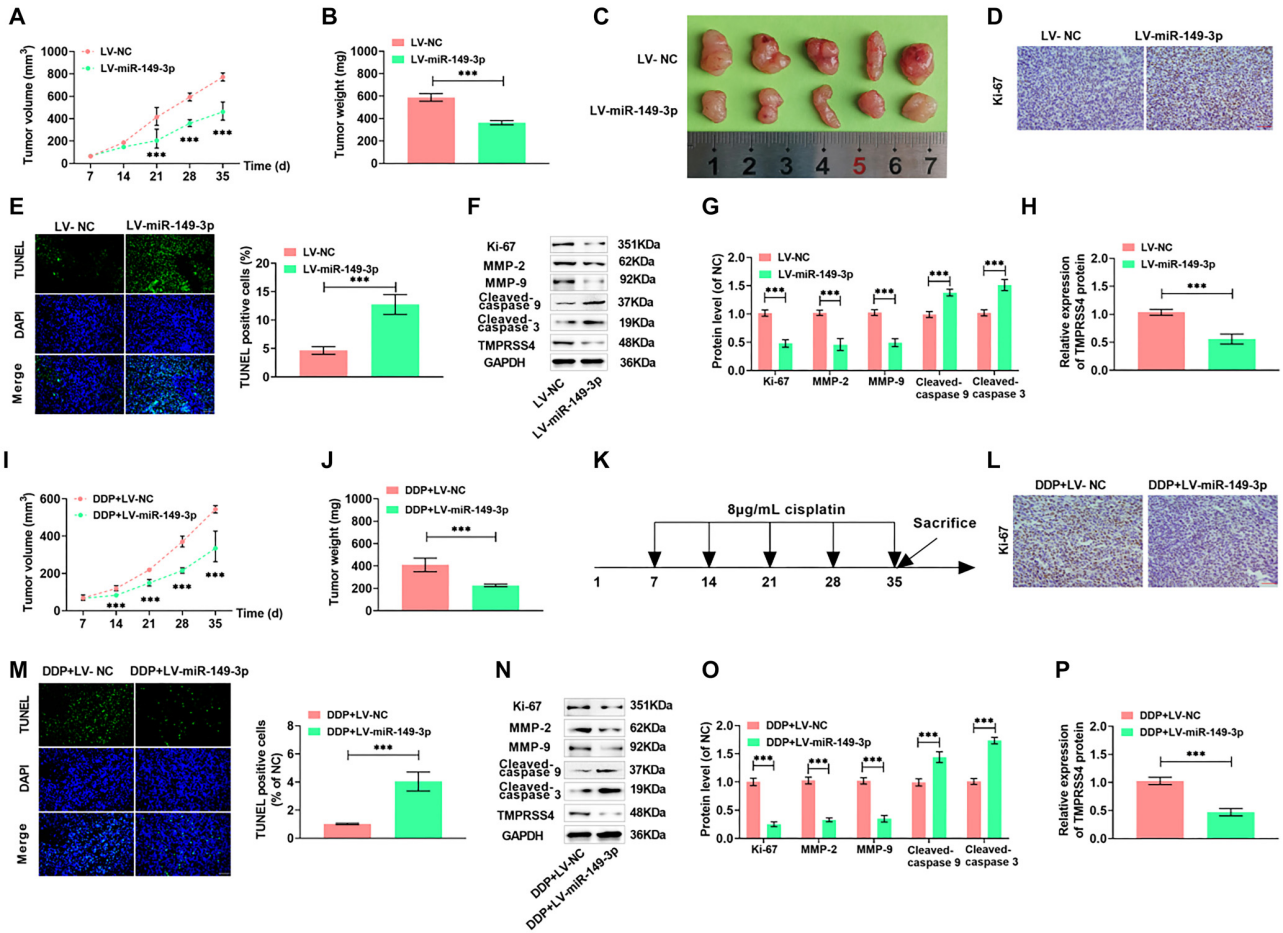
**In vivo experiment**. (A) After subcutaneous inoculation of nude mice with A549 cells, tumor growth was monitored using a vernier caliper at 7-day intervals, starting from day 7 up to day 35; (B and C) At the end of the study, the mice were humanely euthanized, and the tumors were carefully excised for weight measurement and photographic documentation; (D) The proliferation marker Ki67 was evaluated in the excised tumor tissues; (E) Tissue apoptosis was assessed using the TUNEL assay; (F–H) Expression levels of Ki67, Cleaved-caspase 9, Cleaved-caspase 3, MMP-2, MMP-9, and TMPRSS4 proteins were quantified in the tumor samples via Western blot; (I) Following subcutaneous injection of A549 cells into nude mice, the chemotherapeutic agent DDP was administered at a concentration of 8 µg/mL starting on day 7, with tumor size tracked at the same 7-day intervals; (J) The treatment effect was assessed by weighing the tumors on day 35; (K) A schematic representation of the drug administration protocol is provided; (L) Immunohistochemical analysis of Ki67 was performed on tumor tissues; (M) Apoptosis was again evaluated using the TUNEL assay; (N–P) Post-treatment expression levels of Ki67, Cleaved-caspase 9, Cleaved-caspase 3, MMP-2, MMP-9, and TMPRSS4 proteins were reassessed by Western blot. (***P < 0.001) (40×, bar ═ 50 µm). DDP: Diamminedichloroplatinum.

A subcutaneous tumor model was created in nude mice to assess the impact of miR-149-3p overexpression on tumor growth in vivo. Overexpression of miR-149-3p significantly reduced the size and weight of lung cancer tumors, indicating its inhibitory effect on tumor proliferation (Figure 7A–7C). miR-149-3p overexpression also reduced Ki67 levels and increased the number of TUNEL-positive cells (Figure 7D and 7E). Furthermore, miR-149-3p overexpression downregulated Ki67, MMP-2, and MMP-9 proteins while upregulating Cleaved-caspase 9 and Cleaved-caspase 3 proteins, confirming its role in suppressing proliferation and metastasis, and promoting apoptosis in vivo (Figure 7F and 7G). TMPRSS4 expression also decreased significantly, confirming that miR-149-3p targets *TMPRSS4* (Figure 7H). Following DDP injection, miR-149-3p overexpression further reduced tumor volume and weight, enhancing lung cancer cell sensitivity to DDP (Figure 7I and 7J). A schematic of the DDP injection procedure in nude mice is shown in Figure 7K. The combined strategy of miR-149-3p overexpression and DDP chemotherapy led to a greater reduction in Ki67 protein levels and an increase in TUNEL-positive cells (Figure 7L and 7M), as well as reduced Ki67 and MMP-2 expression and further increased Cleaved-caspase 9 and Cleaved-caspase 3 levels, indicating enhanced inhibition of proliferation, migration, and invasion (Figure 7N and 7O). TMPRSS4 expression was further reduced, suggesting that miR-149-3p inhibited lung cancer progression and increased DDP sensitivity by negatively regulating *TMPRSS4* (Figure 7P).

## Discussion

Cancer remains a significant public health challenge in modern society, with a substantial number of deaths occurring annually. Lung cancer, in particular, poses a severe threat to human life and health due to its insidious onset, prolonged latency, short survival time, and high mortality rate [28]. Chemotherapy is often the preferred treatment for most lung cancer patients, as they are typically diagnosed at an advanced stage [29]. DDP, a chemotherapeutic drug known for damaging DNA, has a simple structure. After entering the body, the platinum in DDP crosslinks with one or two DNA strands, forming stable bonds [30]. However, resistance to DDP in lung cancer cells significantly limits its clinical effectiveness and therapeutic outcomes [31]. Thus, uncovering the mechanisms of resistance and enhancing the sensitivity of lung cancer cells to DDP could help address this challenge.

A key regulator in cell growth processes is miRNA, which plays a critical role in the development and progression of tumors, including metastasis, progression, and initiation [32]. Notably, miRNA can also predict drug resistance and DDP sensitivity in lung cancer, offering significant potential for forecasting susceptibility or resistance to DDP [33]. Previous studies have highlighted the role of miR-149-3p in various cancers. For instance, Wang et al. [18] reported that high levels of miR-149-3p in ovarian cancer tissues and DDP-resistant cells result in a poor prognosis and increased DDP resistance. In contrast, Liu et al. observed that colorectal cancer cells exhibit reduced miR-149-3p expression, which mitigates the toxicity of DDP and contributes to the development of DDP resistance [34]. This study focuses on lung cancer. It was found that miR-149-3p is under-expressed in lung cancer cells and tissues. Increasing miR-149-3p levels reduced cell proliferation and enhanced apoptosis, while decreasing its expression led to the opposite effects. Similar findings were reported by Jiang et al. [35], who observed that upregulation of miR-149-3p reduced lung cancer cell proliferation (A549 and H1299) and inhibited tumor growth *in vivo*. Additionally, H446/DDP and A549/DDP cells were developed to examine the role of miR-149-3p in lung cancer’s DDP sensitivity. The expression of miR-149-3p was significantly lower in these DDP-resistant cells compared to normal H446 and A549 cells. Furthermore, overexpression of miR-149-3p impaired cell proliferation and induced apoptosis when treated with DDP. These results confirm that miR-149-3p enhances the sensitivity of lung cancer cells to DDP. To better understand miR-149-3p’s role in increasing the sensitivity of lung cancer cells to DDP, *TMPRSS4* was identified as a potential target gene of miR-149-3p by screening databases such as GEPIA, TargetScan, miRDB, and miRWalk. A dual-luciferase reporter assay confirmed their target relationship. Consistent with Fan et al.’s findings [36], TMPRSS4 was found to be elevated in lung cancer cells and tissues. As demonstrated in other studies [24, 37], downregulation of *TMPRSS4* increases lung cancer cells’ sensitivity to chemotherapeutic drugs. Overexpression of TMPRSS4 partially reversed the inhibitory effects of miR-149-3p overexpression on the proliferation, migration, and invasion of H446, A549, H446/DDP, and A549/DDP cells, while also reducing apoptosis. These findings suggest that the heightened expression of TMPRSS4 weakens the sensitivity of lung cancer cells to DDP. This further supports the idea that miR-149-3p can increase DDP sensitivity in lung cancer cells by specifically targeting *TMPRSS4*. Moreover, *in vivo* experiments confirmed that the combination of miR-149-3p and DDP exerts a stronger regulatory effect on *TMPRSS4*, reinforcing the conclusion that miR-149-3p enhances DDP sensitivity in lung cancer by inhibiting TMPRSS4. This research provides a potential new direction for the clinical management of lung cancer. Developing drugs that target miR-149-3p or *TMPRSS4* could offer an effective approach to halting lung cancer metastasis and improving patient outcomes. However, the study has some limitations. Future research could focus on measuring miR-149-3p levels in lung cancer patients at different stages to explore its relationship with disease progression. Additionally, since DDP resistance in lung cancer is influenced by multiple factors and genes, extensive further research is needed to gain a comprehensive understanding of the regulatory network behind DDP resistance.

## Conclusion

In summary, miR-149-3p expression is significantly reduced in lung cancer tissues and cells. Overexpression of miR-149-3p inhibits cell proliferation, migration, and invasion while promoting apoptosis and increasing DDP sensitivity. This is achieved by specifically silencing *TMPRSS4*, a gene that may promote lung cancer development. Targeting the miR-149-3p/*TMPRSS4* axis shows promise as a therapeutic strategy to overcome chemotherapy resistance in lung cancer patients.

## Supplemental data

**Highlights**

1. miR-149-3p is reduced in lung cancer cells and tissues.
2. Lung cancer cells with elevated levels of miR-149-3p exhibit inhibited proliferation, while their apoptosis and sensitivity to DDP are significantly enhanced.
3. Lung cancer cells exhibit high levels of *TMPRSS4*, which is targeted and downregulated by miR-149-3p.
4. Overexpressed TMPRSS4 promotes cell proliferation, inhibits apoptosis, and partially counteracts the effects of overexpressed miR-149-3p in enhancing the DDP sensitivity of lung cancer cells.
5. Overexpression of miR-149-3p hampers lung cancer progression in nude mice.

**Graphical abstract.**
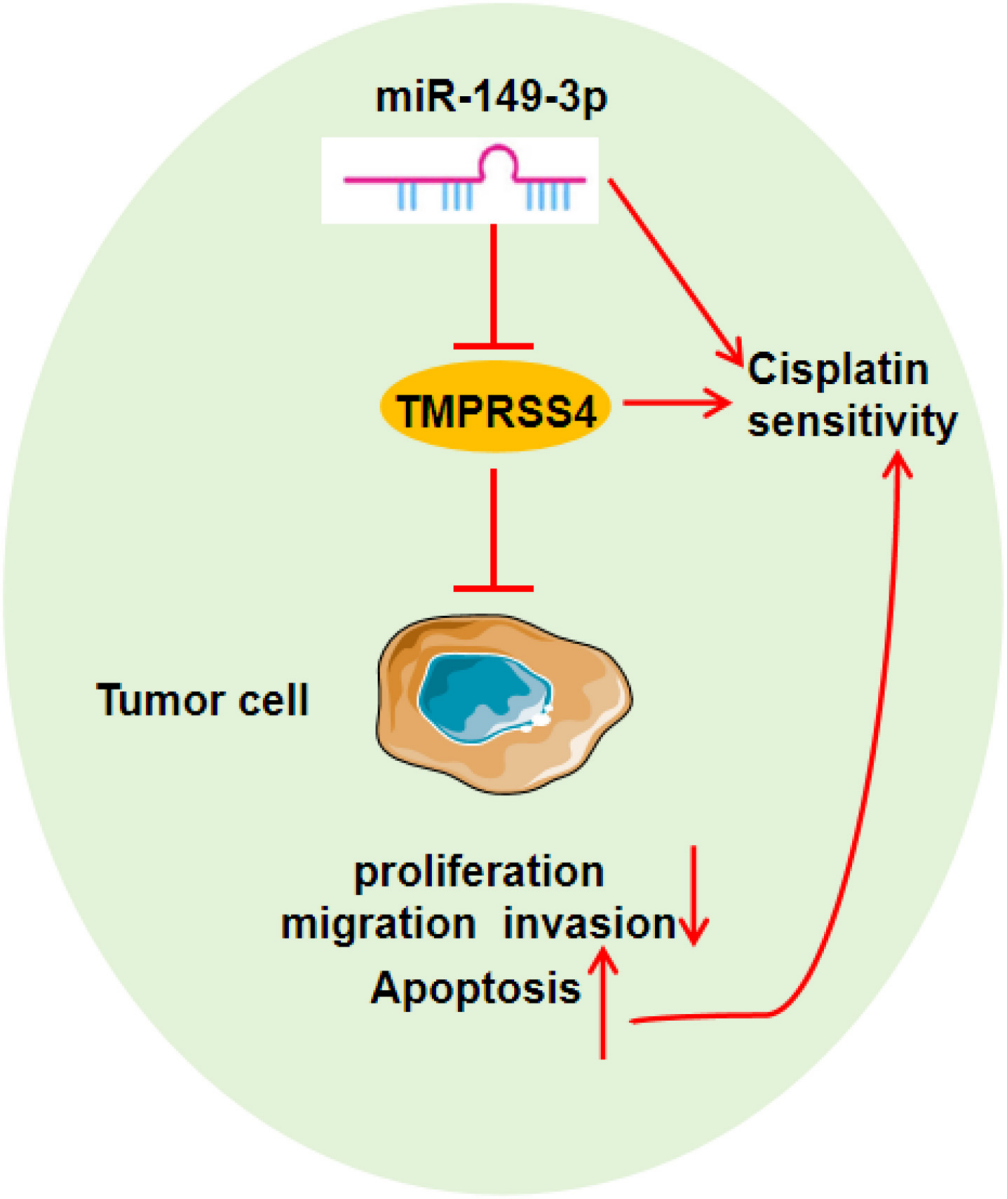
miR-149-3p targeting the *TMPRSS4* mRNA expression enhances the lung tumor cells sensitivity to DDP to restrain the adverse development.

## Data Availability

The data supporting the findings of this study can be obtained from the corresponding author, upon request.
